# *VvJAZ13* Positively Regulates Cold Tolerance in *Arabidopsis* and Grape

**DOI:** 10.3390/ijms25084458

**Published:** 2024-04-18

**Authors:** Lili Che, Shixiong Lu, Huimin Gou, Min Li, Lili Guo, Juanbo Yang, Juan Mao

**Affiliations:** College of Horticulture, Gansu Agricultural University, Lanzhou 730070, China; 18209325789@163.com (L.C.); 18893912407@163.com (S.L.); ghm1648885861@163.com (H.G.); 15002662143@163.com (M.L.); gll0127@163.com (L.G.); 15609437995@163.com (J.Y.)

**Keywords:** grape, overexpression *VvJAZ13*, cold stress, yeast two-hybrid, *CBF*

## Abstract

Cold stress adversely impacts grape growth, development, and yield. Therefore, improving the cold tolerance of grape is an urgent task of grape breeding. The Jasmonic acid (JA) pathway responsive gene *JAZ* plays a key role in plant response to cold stress. However, the role of *JAZ* in response to low temperatures in grape is unclear. In this study, *VvJAZ13* was cloned from the ‘Pinot Noir’ (*Vitis vinefera* cv. ‘Pinot Noir’) grape, and the potential interacting protein of VvJAZ13 was screened by yeast two-hybrid (Y2H). The function of *VvJAZ13* under low temperature stress was verified by genetic transformation. Subcellular localization showed that the gene was mainly expressed in cytoplasm and the nucleus. Y2H indicated that VvF-box, VvTIFY5A, VvTIFY9, Vvbch1, and VvAGD13 may be potential interacting proteins of VvJAZ13. The results of transient transformation of grape leaves showed that *VvJAZ13* improved photosynthetic capacity and reduced cell damage by increasing maximum photosynthetic efficiency of photosystem II (Fv/Fm), reducing relative electrolyte leakage (REL) and malondialdehyde (MDA), and increasing proline content in overexpressed lines (OEs), which played an active role in cold resistance. Through the overexpression of *VvJAZ13* in *Arabidopsis thaliana* and grape calli, the results showed that compared with wild type (WT), transgenic lines had higher antioxidant enzyme activity and proline content, lower REL, MDA, and hydrogen peroxide (H_2_O_2_) content, and an improved ability of scavenging reactive oxygen species. In addition, the expression levels of *CBF1-2* and *ICE1* genes related to cold response were up-regulated in transgenic lines. To sum up, *VvJAZ13* is actively involved in the cold tolerance of *Arabidopsis* and grape, and has the potential to be a candidate gene for improving plant cold tolerance.

## 1. Introduction

Low temperature stress is one of the main environmental factors affecting plant growth and development. The response to low temperature stress and the breeding of cold tolerant plants have attracted wide attention in recent years [1]. Freezing or extremely low temperature is the key factor affecting plant growth and development and crop yield. Plants have evolved a mechanism to increase their tolerance to freezing during exposure to low but non-freezing temperatures, a phenomenon known as cold acclimation [2]. Grape (*Vitis vinifera* L.) is a kind of delicious and nutritious fruit, which has a long history of cultivation and is still one of the fruits with the highest yield in the world. Grape is mainly distributed in arid or semi-arid areas with latitude 25–45° north, and have poor adaptability to the low temperature and arid climate in the main planting areas [3]. Therefore, it is of great practical significance to identify and explore new genes of cold resistance in grape and provide resources for breeding new varieties of cold resistance.

Under low temperature stress, transcription factors (TF) also respond to cold signals. Among them, C-repeat Binding Factor (*CBFs*; also known as dehydration-responsive element-binding proteins or DREBs) transcription factor plays an important role in the response to low temperature stress, and it is identified as the key response factor of plant low temperature stress under cold stress [4,5]. The expression level of the *CBF/DREB1*-like (C-repeat/dehydration-responsive element binding transcription factors) gene *CBF1-4* was increased and maintained at a certain level during low temperature stress. This gene was isolated from the Riparian grape (*Vitis vulpina* L.) and the Eurasian grape, and the analysis suggested that the cold resistance of grape may be related to this gene [6,7]. Among them, the cold resistance of transgenic grape overexpressing *VvCBF4* was significantly improved [8]. In the *CBF*-dependent pathway, CBF protein recognizes the *CRT/DRE* (C repeat/dehydration-responsive element) *cis* element in the promoter region of the *COR* (cold regulated gene, *COR*) gene, which in turn activates the transcription of these downstream genes and leads to cold storage and freezing tolerance in plants. *ICE1* (Inducer of *CBF* expression 1) TF is located in the upstream of the *CBF* gene. Under low temperature stress, *ICE1* can activate the expression of the *CBF3* gene [9]. The activated *CBF* binds to the C-repeat/DRE element on the promoter of the *CORs* gene. For example, heterologous expression of grape *VrCBF1* and *VrCBF4* can promote the expression of *AtCOR6.6*, *AtCOR15a*, and *AtCOR47* genes in *Arabidopsis*, and positively regulate the expression of *AtICE1*, thus enhancing the cold resistance of the plant [10]. The expression of *VvICE1a* and *VvICE1b* improved the cold tolerance of transgenic *Arabidopsis* and led to the up-regulation of stress-related genes *AtRD29A* (Stress-inducible promoter) and *AtCOR47* [11]. In addition, the cold resistance function of transcription factor genes such as *VaERF092* (cold-responsive ethylene response factor), *VaAQUILO* (AcQUIred tolerance to low temperatures), *VbWRKY32* (transcription factors), and *VaPAT1* (a microtubule-interacting protein) in grape was also verified [12,13,14,15].

Recent studies have shown that JA is related to leaf senescence and cold resistance [16]. Exogenous JA promotes leaf senescence and expresses genes related to leaf senescence, thus improving the freezing resistance of *Arabidopsis thaliana*. JA can also improve the cold tolerance of *Arabidopsis* by regulating cold response genes downstream of the *CBF* pathway [17]. *JAZ* is a key gene in the JA pathway. Many negative regulators of the JA signal can be suppressed, such as bHLH (basic helix-loop-helix) subgroup IIId transcription factors [18,19]. During extracellular stimulation or growth and development, JA-Ile enhances the interaction between COI1 (CORONATINE INSENSITIVE 1) and JAZ family proteins, which leads to the ubiquitination and degradation of JAZ by 26S proteasome [20,21]. JAZ proteins belong to a large plant-specific TIFY family. The TIFY family contains a highly conserved ZIM (TIFY) domain. JAZ proteins contain an N-terminal domain and a highly conserved C-terminal Jas domain, which can mediate interactions with *COI1* and multiple transcription factors. Among them, the ZIM domain helps JAZ proteins to form dimers and mediates the interaction between JAZ proteins and co-suppressor NINJA (Noval interactor of *JAZ*). JAZ protein inhibits downstream target genes through Jas domain and negatively regulates the expression of downstream target genes [22,23].

In *Arabidopsis*, JAZ protein directly interacts with MYB21 and MYB24, which can regulate the model of JA biosynthesis to trigger *COI1*-dependent *JAZ* degradation to control MYB21 and MYB24 levels, thereby controlling stamen development [24]. In *Arabidopsis*, AtJAZ3 targeting proteins TOE2 (TARGET OF EAT 2) and KAN1 (KANADI1) interact directly with the inhibitor TPL (TOPLESS). These *JAZ* targets seem to be mainly transcription factors related to the hormone regulation of plant development and light signal transduction [25]. Photosensitive pigments and JAZ proteins are involved in the interaction between light and JA signals, thus affecting plant photosynthesis [26]. The coordination of JA signal crosstalk may be one of the important functions of JAZ protein. Recent studies have shown that rice (*Oryza*) *JAZ9* plays a key role in mediating crosstalk between JA and gibberellin (GA). It interacts with DELLA protein (SLENDER RICE 1). Overexpression of *OsJAZ9* enhanced the response of rice to GA [27]. In fact, JA is a positive regulator of leaf senescence because JAZ7 protein inhibits dark-induced leaf senescence, while *MYC* transcription factors promote senescence by activating senescence-related genes and chlorophyll degradation-related genes, indicating that JA stimulates leaf senescence in a COI1-dependent manner. In this pathway, *JAZ4*, *JAZ8*, and *WRKY57* act as negative regulatory factors, while *IAA29*, an auxin signal transduction inhibitor, acts as a positive regulatory factor in contrast to *JAZ7* [28]. *JAZ* also inhibits the transcriptional activity of ETHYLENE-INSENSITIVE 3 (EIN3)/EIN3-LIKE (EIL) downstream of APETALA2/ETHYLENE RESPONSE FACTOR (AP2/ERF) domain transcription factor *ORA59* (*ORA59/ERF1*) gene in ethylene pathway and induces its expression, thus resisting the infection of necrotizing pathogens and nutritional pathogens [29].

It is found that the expression of the *JAZ* gene is regulated by various abiotic stresses such as cold, saline-alkali, drought and so on. In mosses (*Racomitrium canescens*), overexpression of the *JAZ1* gene can enhance salt stress tolerance and inhibit the expression of genes related to abscisic acid (ABA) pathway [30]. In peppermint (*Mentha Canadensis*) leaves and roots, the expression of *McJAZ8* was up-regulated to varying degrees under drought and NaCl treatments, indicating that *McJAZ8* was involved in the response of peppermint to severe cold and salt stress [31]. In *Arabidopsis*, the *JAZ* gene enhances the drought tolerance of *Arabidopsis* by regulating redox, plant hormones, and secondary metabolites [32]. In rice, overexpression of *OsJAZ1* can enhance the drought resistance of rice, and overexpression of *OsJAZ9* can enhance salt and alkali tolerance of rice plants [17]. The expression of the *GhJAZ1* gene in cotton (*Gossypium hirsutum* L.) was up-regulated under low temperature stress, indicating that the gene was involved in the response to low temperature stress [33]. The heterologous expression of soybean (*Glycine max*) *GsJAZ2* gene in *Arabidopsis* can enhance the tolerance of *Arabidopsis* to saline-alkali stress [34]. In all, these studies show that the JAZ gene family plays a positive role in plant response to cold stress, and *VvJAZ13* may be an important candidate gene to improve stress resistance in grape molecular breeding.

In this study, we identified *VvJAZ13* from the ‘Pinot Noir’ grape. The effect of *VvJAZ13* on cold stress was examined in transgenic *Arabidopsis* and grape calli. In addition, we also studied the interaction protein of *VvJAZ13* in grape and the physiological and biochemical indexes of *Arabidopsis* and grape calli overexpressed by *VvJAZ13* under cold stress.

## 2. Results

### 2.1. Cloning and Phylogenetic Analysis of the VvJAZ13 Gene

**Figure 1:**
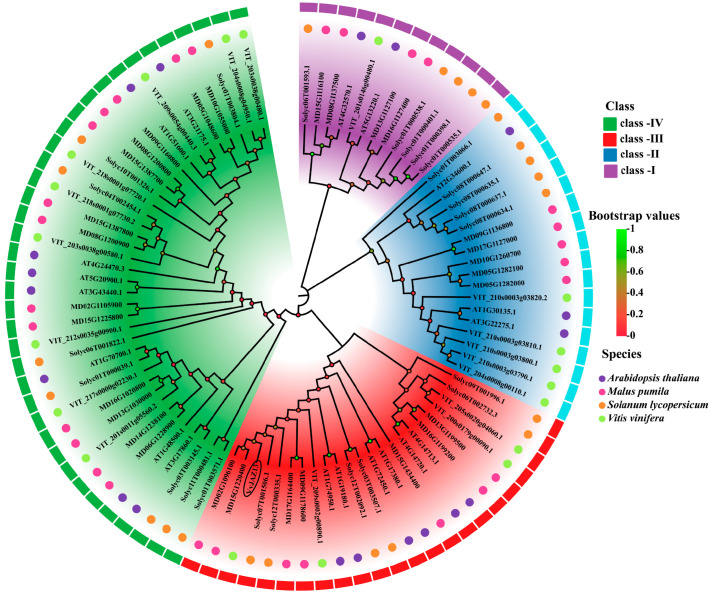
Evolutionary analysis of the *VvJAZ13* gene in grape.

According to the previous transcriptome data, in the experiment, we cloned the *GSVIVG01015042001* gene from the ‘Pinot Noir’ grape and renamed it *VvJAZ13* on the basis of cloning and chromosome mapping of the grape gene (Figures S1 and S2). Based on the phylogenetic tree construction of the grape, *Arabidopsis*, apple, and tomato JAZ gene family members (Figure 1), these four species can be divided into four subfamilies. The *VvJAZ13* gene clustered in III subgroup and is closely related to apple and tomato.

### 2.2. Transient Expression of VvJAZ13 in Tobacco

**Figure 2:**
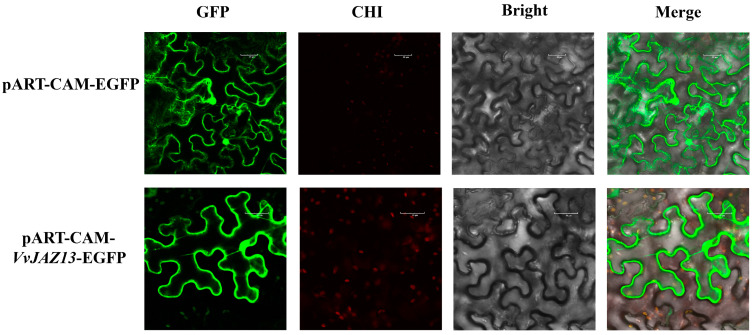
Subcellular localization of VvJAZ13 protein. The GFP fluorescence image, Chloroplast auto fluorescence (CHI) image, bright field (Bright) image, and the merged GFP, CHI, and Bright images are shown. Scare bar: 30 μm.

We constructed the pART-CAM-*VvJAZ13*-EGFP vector. The empty plasmids of pART-CAM-*VvJAZ13*-EGFP and pART-CAM-EGFP were transformed into *Agrobacterium tumefaciens* GV3101 and injected into tobacco leaves. The results showed that VvJAZ13 was localized in the cytoplasm and nucleus (Figure 2).

### 2.3. The Interaction Proteins Screening of VvJAZ13

According to the CDS sequence of *VvJAZ13*, pGBKT7-*VvJAZ13* primers were designed and amplified (Table S1). The pGBKT7-*VvJAZ13* recombinant plasmid was introduced into yeast receptor cells Y2H gold (Figure S3), which were coated on SD/-Trp and SD/-Trp/-Leu/-His plates, respectively, and was found not to grow on SD/-Trp/-Leu/-His plates, suggesting that no self-activation was detected for VvJAZ13 (Figure S4).

Using pGBKT7-VvJAZ13 as bait protein, the monoclonal strain was screened on an SD/-Trp/-Leu/-His/-Ade plate by Mating double hybridization. Through blast comparison in NCBI, it was found that most of the genes were related to abiotic stress. For example, VvF-box (core subunit of E3 ubiquitin ligase SCF complex), VvTIFY5A (JA signal regulatory protein), VvTIFY9 (JA signal regulatory protein), Vvbch1 (β-carotene hydroxylase), VvAGD13 (ADP-ribose factor GTP enzyme activating protein AGD13) (Figure S5).

**Figure 3:**
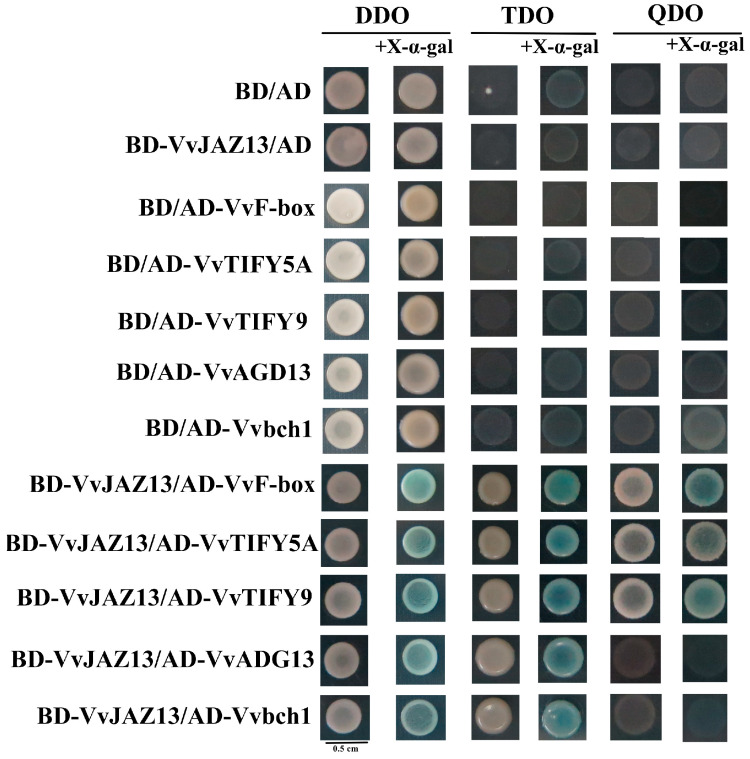
Verification of interaction between VvJAZ13 protein and interaction protein in the yeast two-hybrid system. AD and BD represent pGBKT7 and pGADT7, respectively. DDO indicates SD/-Trp, TDO indicates SD/-Trp/-Leu/-His, QDO indicates SD/-Trp/-Leu/-His/-Ade, X indicates X-α-gal.

The target gene fragments of the same size were obtained by PCR amplification with pGADT7-VvF-box, pGADT7-VvTIFY5A, pGADT7-VvTIFY9, pGADT7-Vvbch1, and pGADT7-VvAGD13 primers (Table S1 and Figure S6). The recombinant plasmids BD-VvJAZ13/AD-VvF-box, BD-VvJAZ13/AD-VvTIFY5A, BD-VvJAZ13/AD-VvTIFY9, BD-VvJAZ13/AD-Vvbch1, and BD-VvJAZ13/AD-VvAGD13 were co-transformed into Y2Hgold yeast competent cells and verified. BD/AD, BD-VvJAZ13/AD, BD/AD-VvF-box, BD/AD-VvTIFY5A, BD/AD-VvTIFY9, BD/AD-Vvbch1, and BD/AD-VvAGD1 co-transduction was used as control. They were spread on DDO, DDO + X, TDO, TDO + X, QDO, and QDO + X plates, respectively. It was found that BD-VvJAZ1/AD-VvF-box, BD-VvJAZ1/AD-VvTIFY5A, and BD-VvJAZ1/AD-VvTIFY9 grew and turned blue in QDO + X, indicating that there was an interaction between VvJAZ13 and VvF-box, VvTIFY5A, and VvTIFY9. BD-VvJAZ1/AD-Vvbch1 and BD-VvJAZ1/AD-VvAGD13 did not grow in QDO but grew and turned blue in TDO + X, indicating that there was a weak interaction between VvJAZ13 and Vvbch1 and VvAGD13 (Figure 3).

### 2.4. The Instantaneous Transformation of VvJAZ13 Improved the Cold TOLERANCE of Grape Leaves

**Figure 4:**
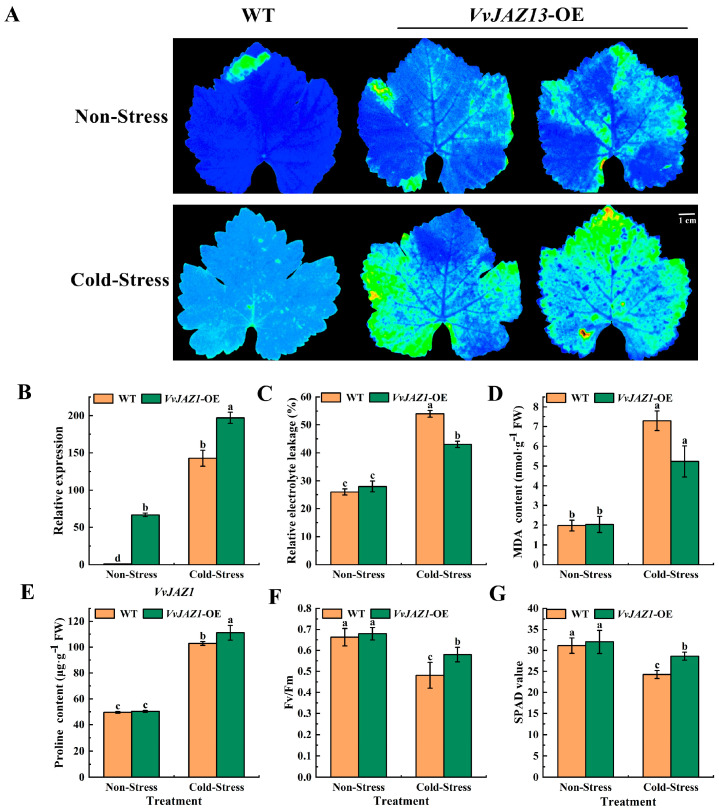
Transient transformation of *VvJAZ13* in grape leaves. (**A**) Fluorescence intensity of WT and *VvJAZ13*-OEs grape leaves, (**B**) Expression of *VvJAZ13* in WT and *VvJAZ13*-OEs grape leaves, (**C**–**E**) REL, MDA, and proline contents of WT and *VvJAZ13*-OEs grape leaves (**F**) Fv/Fm of WT and *VvJAZ13*-OEs grape leaves, (**G**) SPAD value of WT and *VvJAZ13*-OEs grape leaves. Based on Duncan’s multiple range test, lowercase letters represent significant differences (*p* < 0.05).

Gene *VvJAZ13* was overexpressed in grape leaves by the *Agrobacterium*-mediated transient transformation system to characterize the gene function under cold stress. The results showed that the fluorescence intensity of grape leaves overexpressing *VvJAZ13*-OE was significantly higher than that of WT grape leaves under cold-stress treatment (Figure 4A).

Under non-stress treatment, the transcriptional level of *VvJAZ13*-OE grape leaves was higher than that of WT grape leaves, indicating that *VvJAZ13* was successfully transformed into grape leaves. Under cold-stress treatment, the expression of *VvJAZ13* in overexpressed grape leaves was significantly higher than that in WT grape leaves (Figure 4B). Thereafter, the *VvJAZ13*-OE and WT leaves were exposed to 4 °C for 24 h. Consistent with fluorescence intensity, REL and MDA contents in *VvJAZ13*-OE grape leaves decreased by 20.37% and 28.57% compared with WT leaves, respectively, while proline contents increased by 8.82% compared with WT leaves (Figure 4C–E). Compared with WT leaves, the Fv/Fm and SPAD values of *VvJAZ13*-OE leaves increased by 20.83% and 24.26%, respectively (Figure 4F,G).

Subsequently, the transcript abundances of *VvCBF1*, *VvCBF2*, *VvCBF3*, *ICE1a*, *ICE1b*, and *ICE1c* were examined. The results showed that *VvJAZ13* overexpression promoted the expression of *VvCBF* and *VvICE1* genes, especially the *VvCBF3* gene under cold stress, which was 38.02% higher than that of WT. These results indicated that overexpression of *VvJAZ13* improved the cold resistance of the grape leaves (Figure S7).

### 2.5. Overexpression of VvJAZ13 Increased Cold Tolerance in Transgenic Arabidopsis

The transformed *Arabidopsis* seeds were screened on the MS medium containing Kana, and the *Arabidopsis* lines with overexpression of *VvJAZ13* were obtained (Figure S8A,B). Three transgenic lines OE-1, OE-2, and OE-3 were selected for functional verification (Figure S8C).

**Figure 5:**
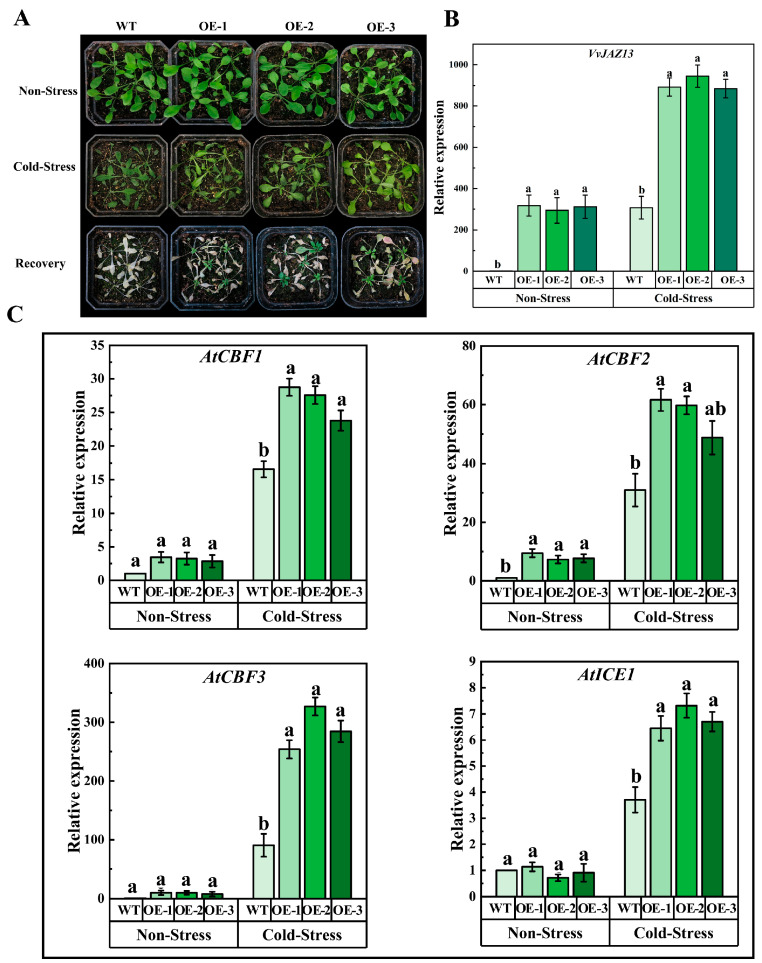
Phenotypic aspect and gene expression levels of *VvJAZ13* in *Arabidopsis* transgenic lines under cold stress. (**A**) Phenotypes of WT and *VvJAZ13*-OEs *Arabidopsis*, (**B**) Expression levels of the *VvJAZ13* gene in WT and *VvJAZ13*-OEs *Arabidopsis*, (**C**) Expression levels of the cold response genes *AtCBF1*, *AtCBF2*, *AtCBF3*, and *AtICE1* in WT and *VvJAZ13*-OEs *Arabidopsis*. The bar represents the value of the standard error (three biological replicates). Based on Duncan’s multiple range test, lowercase letters represent significant differences (*p* < 0.05).

The results showed that cold stress of WT was more serious than that of OEs. One week after recovery, the survival rate of OEs was significantly higher than that of WT (Figure 5A). qRT-PCR analysis of *Arabidopsis* showed that the expression of the *VvJAZ13* gene was significantly up-regulated under cold stress treatment (Figure 5B).

Analysis of the changes of cold response genes *AtCBF* and *AtICE1* in transgenic *Arabidopsis* is shown in Figure 5C. The results showed that there was no significant difference in the expression of *AtCBF1*, *AtCBF2*, *AtCBF3*, and *AtICE1* between transgenic *VvJAZ13 Arabidopsis* and WT before stress. However, under cold stress, the expression of *AtCBF1*, *AtCBF2*, and *AtCBF3* in transgenic *Arabidopsis* was significantly higher than that in WT; especially, the expression of *AtCBF3* in OEs increased by 181.11%, 262.22%, and 215.56%, respectively, compared with WT (Figure 5C). The results indicated that overexpression of *VvJAZ13* can lead to upregulation of *AtCBF1-3* and *AtICE1* gene expression in *Arabidopsis* under cold stress conditions.

**Figure 6:**
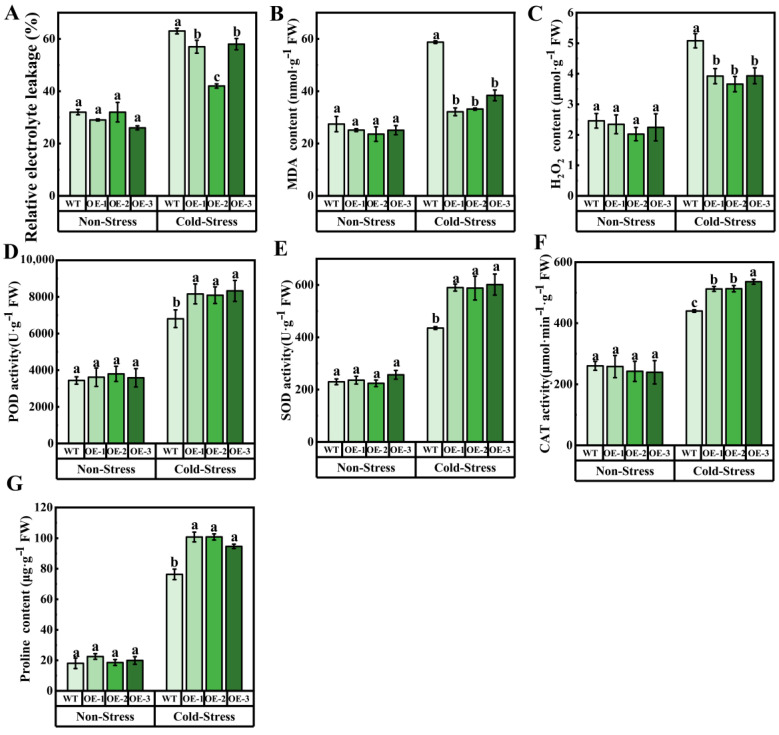
Analysis of membrane lipid peroxidation and antioxidant enzyme activities of *Arabidopsis* transgenic lines of *VvJAZ13* under cold stress. (**A**) REL of WT and OEs *Arabidopsis*, (**B**) MDA content of WT and OEs *Arabidopsis*, (**C**) H_2_O_2_ content of WT and OEs *Arabidopsis*, (**D**–**F**) POD, SOD, and CAT activities of WT and OEs *Arabidopsis*, (**G**) Proline content of WT and OEs *Arabidopsis*. The bar represents the value of the standard error (three biological replicates). Based on Duncan’s multiple range test, lowercase letters represent significant differences (*p* < 0.05).

At the same time, under cold stress, the REL and MDA content in OE in OE were lower than those in WT, indicating that the damage to cell membrane caused by cold stress in transgenic *Arabidopsis* was reduced. In addition, compared with WT, the H_2_O_2_ content of transgenic *Arabidopsis* decreased under cold stress, while the activity of antioxidant enzymes (CAT, SOD, POD) and proline content increased, of which proline content increased by 31.58%, 32.70%, and 24.55%, respectively, compared with WT (Figure 6). To sum up, overexpression of *VvJAZ13* increased the ability of scavenging reactive oxygen species (ROS), reduced the degree of membrane lipid peroxidation and cell dehydration, and increased the expression of cold response genes to protect *Arabidopsis* from cold stress.

### 2.6. Overexpression of VvJAZ13 Increased Cold Tolerance in Transgenic Grape Calli

**Figure 7:**
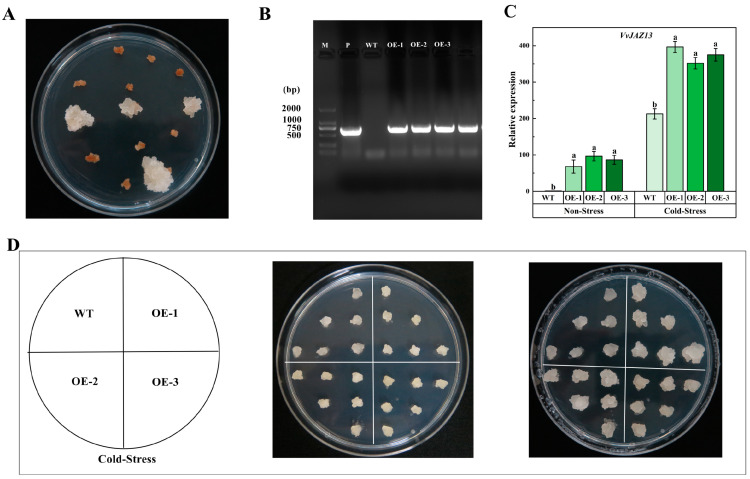
*VvJAZ13* identification of transgenic grape calli. (**A**) Screening of transgenic calli in a B_5_ medium containing Kana resistance, (**B**) Positive detection of grape calli, (**C**) Expression levels of the *VvJAZ13* gene in WT and *VvJAZ13*-OEs grape calli, (**D**) Phenotypes of WT and *VvJAZ13*-OEs grape calli. The bar represents the value of the standard error (three biological replicates). Based on Duncan’s multiple range test, lowercase letters represent significant differences (*p* < 0.05).

After screening on the medium containing 10 mg·L^−1^ kanamycin, new growth of resistant calli was observed (Figure 7A). The new calli were transferred to the same screening medium again. After 30 days of culture, DNA was extracted and PCR was performed. The results showed that they were transformed successfully (OE-1, OE-2, OE-3) (Figure 7B). After cold stress at 4 °C for 10 days, it was found that OE grew faster than WT calli (Figure 7D). The results of qRT-PCR showed that compared with WT, the expression of *VvJAZ13* was significantly up-regulated in transgenic calli under cold stress (Figure 7C).

**Figure 8:**
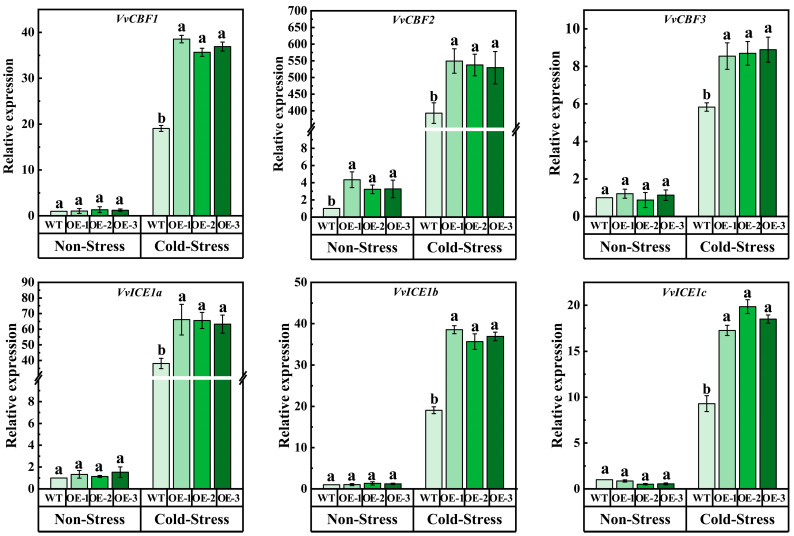
Expression levels of *VvCBF1*, *VvCBF2*, *VvCBF3*, *VvICE1a*, *VvICE1b*, and *VvICE1c* in WT and *VvPAL10*-OEs grape calli. The bar represents the value of the standard error (three biological replicates). Based on Duncan’s multiple range test, lowercase letters represent significant differences (*p* < 0.05).

In order to further study the mechanism of potential cold tolerance of *VvJAZ13* overexpression, the transcriptional abundance of cold-related genes (*VvCBF1*, *VvCBF2*, *VvCBF3*, *VvICE1a*, *VvICE1b*, and *VvICE1c*) was determined. Under non-stress treatment, the expression levels of 6 genes were similar between WT and OE. However, under cold treatment, the expression of these genes in *VvJAZ13* overexpression plants was significantly higher than that in WT plants, especially that in *VvCBF2* was significantly higher than that in WT, which was 39.71%, and 34.63% higher than that in WT, respectively (Figure 8).

**Figure 9:**
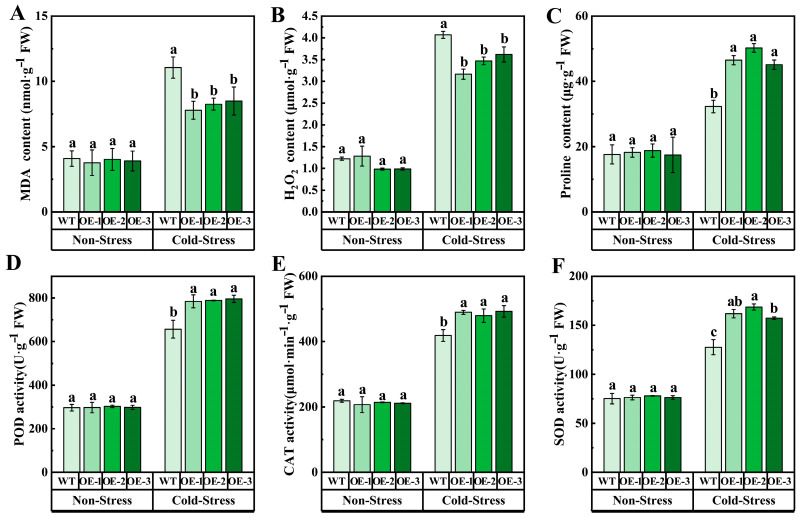
Analysis of membrane lipid peroxidation and antioxidant enzyme activities of grape calli transgenic lines of *VvJAZ13*. (**A**) The content of MDA in WT and *VvPAL10*-OEs grape calli, (**B**) The content of H_2_O_2_ in WT and *VvPAL10*-OEs grape calli, (**C**) The content of Proline in WT and *VvPAL10*-OEs grape calli, (**D**–**F**) POD, SOD, and CAT enzymes activities were determined in WT and VvPAL10-OEs grape calli. The bar represents the value of the standard error (three biological replicates). Based on Duncan’s multiple range test, lowercase letters represent significant differences (*p* < 0.05).

In order to study the effect of *VvJAZ13* overexpression on grape calli under low temperature treatment, the physiological indexes of WT and OEs were further determined. The results showed that there was no significant difference in MDA and H_2_O_2_ contents between WT and OEs under non-stress, but the MDA and H_2_O_2_ contents of WT were significantly higher than those of OEs under cold stress. In order to alleviate the high level of ROS, the antioxidant mechanisms such as SOD, POD, and CAT were activated and the content of proline increased [35]. Under non-stress conditions, there was no significant difference in SOD, POD, CAT, activity and proline content between transgenic calli and WT, but the antioxidant enzyme activity and proline content of transgenic calli after cold treatment were higher than those of WT. The activity of POD increased by 19.45%, 20.12%, and 21.17%, and the content of proline increased by 43.90%, 55.54%, and 39.69% compared with WT (Figure 9). These results suggest that overexpression of *VvJAZ13* enhances cold tolerance of grape calli by inhibiting severe cell membrane damage and activating antioxidant mechanism.

## 3. Discussion

Cold stress will adversely affect the growth and development of plants. Most temperate plants acquire cold tolerance through a process called cold adaptation. Cold stress is an important environmental restriction, which greatly affects plant growth and productivity and limits plant geographical distribution [1]. About 51% to 82% of crop yield losses are caused by extreme cold stress [36]. Under cold stress, plants will undergo a series of damage, including the destruction of cell membrane and cell structural stability, the decrease in photosynthesis and metabolism, and the excessive production of ROS [37,38,39].

In tea (*Camellia sinensis*), the CsJAZ1-1 fluorescence signal is only located in the nucleus, while CsJAZ1-2 and CsJAZ1-3 are widely distributed in the cytoplasm and nucleus. When these plasmids were transformed into *Arabidopsis* protoplasts, similar results were obtained. In short, the results showed that the subcellular localization of the three CsJAZ1 subtypes was determined by the N-terminal and Jas domain [40]. In this study, the transient transformation of tobacco leaves showed that VvJAZ13 was widely distributed in the cytoplasm and nucleus, which was the same as the mapping of CsJAZ1-2 and CsJAZ1-3 genes in tea.

JAZ proteins are a family widely involved in plant metabolism, defense and development [22]. Recently, the role of the JAZ protein family in the molecular mechanism related to the response to low temperature stress has been studied. For example, the interaction between *JAZ1* and *JAZ4* in *Arabidopsis* inhibits the transcription factors *ICE1* and *ICE2*, thus inhibiting the *ICE1-CBF/DREB1* signal pathway [16]. The JAZ signaling pathway leads to the release of *bHLH* and *MYB* TFs, which promote anthocyanin accumulation in *Arabidopsis thaliana*, thus improving its cold tolerance [41]. Plants have formed a complex regulatory mechanism against cold stress. In particular, the *CBF* cold response pathway plays a vital role in enhancing cold tolerance by directly activating multiple cold response genes [42,43]. In *Arabidopsis*, *AtCBF1*-*AtCBF3* has been identified, and the target gene of *CBF* has been reported [44]. As a cold response gene, the *CBF* gene can be induced rapidly by low temperature. Chilling is mediated by a variety of transcription factors. For example, *CBF* expression inducer 1 (*ICE1*) can activate the expression of *CBF*, thereby enhancing the tolerance of plants to low temperature stress [45]. In our study, the relative expression levels of *AtCBF1*, *AtCBF2*, *AtCBF3*, and *AtICE1* in transgenic *Arabidopsis* were up-regulated under cold stress. At the same time, the transcriptional abundance of *VvCBF1*, *VvCBF2*, *VvCBF3*, *VvICE1a*, *VvICE1b*, and *VvICE1c* genes in *VvJAZ13* transgenic grape calli also increased. In addition, the expression of *VvCBF* and *VvICE1* genes (*VvCBF1*, *VvCBF2*, *VvCBF3*) in *VvJAZ13* transient overexpression transgenic grape leaves was also upregulated under cold stress. Therefore, we speculate that *VvJAZ13* may respond to cold stress by directly regulating *CBF* signaling transduction in grape.

In kiwifruit (*Actinidia arguta*), the overexpression of *AaBAM3.1* lines showed increased freezing tolerance with higher chlorophyll content and chlorophyll fluorescence [46]. We speculate that low temperature may inhibit the synthesis of chlorophyll. Overexpressed *VvJAZ13* had a slightly lower sensitivity to inhibition, increased Fv/Fm and SPAD, and a higher efficiency of chlorophyll synthesis, which maintained the ability of the photosynthetic system, thus enhanced the cold tolerance of grape leaves.

The level of REL is considered to be the analysis of plant cell membrane damage under abiotic stress [47]. Meanwhile, MDA, as the final product of lipid peroxidation, is widely used as a marker of ROS-mediated plant damage [48]. In this study, *VvJAZ13* overexpression significantly decreased REL in *Arabidopsis* and grape leaves. The content of MDA also decreased significantly in *Arabidopsis* and grape calli with *VvJAZ13* overexpression. It is suggested that the overexpression of *VvJAZ13* can prevent cell membrane damage caused by cold stress and respond to low temperature. Under low temperature stress, oxidative damage caused by excessive ROS accumulation is harmful to cell function and biological processes [45]. Under normal growth environment, there is a dynamic balance between ROS production and scavenging. However, when plants are subjected to cold stress, ROS production increases significantly, while antioxidant defense systems reduces the effects of oxidative stress on plants [49,50,51]. Proline accumulation is one of the important physiological responses of plants to resist low temperature stress, and plays an important role in plant cold resistance [52]. In our study, *VvJAZ13* overexpression in *Arabidopsis* could significantly increase the activity and proline content of POD, SOD, CAT, and proline compared with WT under cold treatment. Similarly, under cold stress, we found that compared with WT calli, the overexpression of *VvJAZ13* increased the activities of SOD, POD, CAT, and the content of proline. Therefore, we speculate that *VvJAZ13* can reduce the accumulation of ROS by increasing the activity of antioxidant enzymes, so as to reduce cell membrane damage, reduce cell death, and enhance the cold resistance of grape. These results indicated that *VvJAZ13* overexpression could improve the antioxidant capacity of *Arabidopsis* and grape, and thus enhance the cold resistance.

Plants have evolved complex regulatory mechanisms to cope with the effects of the external environment. The ubiquitin proteasome pathway involved in F-box is one of the key biological regulatory pathways, and a few studies have shown that F-box protein plays an important role in responding to abiotic stresses such as drought, salt, and temperature stress in plants. For example, the expression of F-box protein 7 in *Arabidopsis* was induced by low temperature stress, and the protein biosynthesis of *fbp7* mutants was inhibited under low temperature stress [53]. The interaction between the F-box family gene ShPP2-1 (phloem protein 2-1) and ACR11A (ACT DOMAIN REPEAT) regulates the cold tolerance of tomato (*Solanum lycopersicum*) [54]. Overexpression of rice F-box protein *MAIF1* (miRNAs regulated and abiotic stress induced F-box gene) reduces abiotic stress tolerance and promotes root growth, and may play a negative role in response to abiotic stress by regulating root growth [55]. TIFY proteins are a class of transcription factors unique to plants, characterized by highly conserved TIFY domains, which play important regulatory roles in plant growth and development and stress tolerance [56]. Overexpression of *AtTIFY10a*, *10b*, and *GsTIFY10a* in *Arabidopsis* (as its wild soybean homolog) and *OsJAZ8* in rice significantly regulated plant response to alkaline and salt stress, respectively [57,58]. The TIFY gene family has multiple regulatory roles in cell signal transduction and regulation of plant response to stress, so it may be a valuable stress response gene resource. The β-carotene hydroxylase gene *bch1* can catalyze β-cryptoxanthin to synthesize zeaxanthin, which plays an important role in plant resistance to abiotic stress [59]. In this study, a yeast double hybrid showed that VvF-box, VvTIFY5A, VvTIFY9, and Vvbch1 had potential interaction with VvJAZ13 and played an important role in cold resistance of grape. However, we have not verified its interaction, and the interaction mechanism between grape and *VvJAZ13* in cold resistance is not clear, and subsequent experiments are needed to study its molecular mechanism.

## 4. Materials and Methods

### 4.1. Test Material and Treatment

‘Pinot Noir’ grape plantlets were preserved in the plant tissue culture room of the Horticulture College of Gansu Agricultural University (Lanzhou, China). Tobacco (*Nicotiana benthamiana*) and *Arabidopsis* (ecotype Columbia) seeds were purchased from Nanjing Fengshuo Horticulture Ltd., Company (Nanjing, China). ‘Pinot Noir’ grape plantlets were subcultured in a GS solid medium (improved B_5_ solid medium) and cycled under 28 °C/25 °C and 12,000 lx light (LED plant lamp) for 16 h/dark for 8 h. After 35 days of culture, half of the samples were subjected to cold stress at 4 °C in a low-temperature incubator for 3 days.

‘Pinot Noir’ grape calli was preserved in a dark incubator at 28 °C. The culture conditions of *Arabidopsis* was 28 °C/25 °C, light for 16 h/dark for 8 h, and light intensity of 12,000 lx (LED plant lamp).

Tobacco was cultured in an incubator with 22 °C/18 °C, light intensity 10,000 lx (LED plant lamp), light for 16 h/dark for 8 h.

### 4.2. Chromosome Mapping and Evolutionary Analysis of VvJAZ13

The protein sequences of *JAZ* genes were obtained from the plant genome database phytozome v13.1: Home (https://phytozome-next.jgi.doe.gov/, accessed on 11 October 2023). Phylogenetic tree was constructed in MEGA 7.0 using the Neighbor Joining Algorithm (NJ) and set up with 1000 bootstrap repetitions [60]. The chromosome location was visualized by the software TBtools (version 1.108).

### 4.3. RNA Extraction and qRT-PCR

Total RNA was extracted from grape, *Arabidopsis* plant leaves, and grape calli by the RNAprep Pure Plant Kit (TIANGEN, Beijing, China). All primers were designed on an online website and synthesized by Sangon Biotech Co., Ltd. (Shanghai, China) (Table S1). The *GAPDH* gene (GenBank No. CB973647) was used as an internal control gene. Total reaction volume was 20 μL, including 7 μL ddH_2_O, 1 μL cDNA, 2 μL upstream and downstream primers, and 10 µL TaKaRa SYBR Premix Ex Taq. II (TaKaRa Biotechnology, Lanzhou, China). The cycling parameters were 95 °C for 30 s, 40 cycles of 95 °C for 5 s, and 60 °C for 34 s. Melting curve analysis was performed after the PCR cycle, with a program that included 95 °C for 15 s, 60 °C for 60 s, and 95 °C for 15 s [61].

### 4.4. Gene Cloning and Subcellular Location

The CDS of the *VvJAZ13* (*GSVIVG01015042001*) gene was amplified by the homologous recombination technique. Primers were designed according to the CDS sequence in ‘Pinot Noir’(PN40024) 12Xv1 genome accession. Primers were synthesized by Sangon Biotech (Shanghai, China) Co., Ltd. (Table S1). Subcellular localisation of *VvJAZ13* was performed with reference to Gou et al. [62]. The specific steps were as follows: the positive *Agrobacterium* containing the candidate gene was activated and cultured, and then expanded and centrifuged to collect the bacterial cells after activation, and then resuspended in a resuspension solution (10 m mol·L^−1^ MES, 10 mmol·L^−1^ MgCl_2_, 150 μmol·L^−1^ Acetosyringone) adjust to OD_600_ = 0.75, using the disposable syringe prepared in advance to accurately inject the bacteria liquid from the back of the prepared tobacco leaves, so that all the bacteria liquid could penetrate into the leaf flesh of the tobacco leaves. The injected tobacco was cultured in the dark for 24 h, and then GFP fluorescence was detected by a laser confocal scanning microscope (Olympus FV1000 Viewer, Tokyo, Japan) to observe the expression of *VvJAZ13*.

### 4.5. Instantaneous Transformation of Grape Leaves

The *Agrobacterium tumefaciens* solution was put into a 10 mL centrifuge tube and centrifuged for 5 min at 6000 rpm. The supernatant was removed and the heavy suspension was added to OD_600_ = 0.4–0.6. The suspension was activated for 3 h and infected grape leaves. The transient expression of grape leaves mediated by *Agrobacterium tumefaciens* refers to the method of Li et al. [63]. The transfected leaves were cultured at 25 °C for 24 h, then some of them were cultured at 4 °C and 16 h/8 h photoperiod for 24 h, and some of them were cultured at 25 °C and 16 h/8 h photoperiod for 24 h. The chlorophyll fluorescence imaging system (IMAPING-PAM, Walz, Rohrdorf, Germany) was used for visualization and determination of Fv/Fm. The SPAD value was determined by a chlorophyll content tester (SPAD-502Plus, Konica Minolta, Tokyo, Japan).

### 4.6. Arabidopsis Thaliana Transformation and Treatment

*Arabidopsis* was transformed by the *Agrobacterium tumefaciens*-mediated flower soaking method to obtain T_0_ generation transgenic plants [64], The T_1_ generations of positive transgenic seeds were selected on MS + 3% sucrose medium containing 50 mg·L^−1^ kanamycin, and the live seedlings were transplanted in nutritious soil to identify transgenic lines and harvest seeds. The same method was repeated to screen T_2_ and T_3_ generations. Finally, the T_3_ generation plants of three *Arabidopsis* lines (OE-1, OE-2 and OE-3) overexpressing *VvJAZ13* were treated with cold stress.

Three stable T_3_ transgenic lines were screened from *Arabidopsis*. Some 3-week *Arabidopsis* plants with good growth and consistent growth, including transgenic plants (OEs) and wild type (WT) plants, were selected and placed in the plant low temperature incubator. It was cooled gradually from 24 °C to 4 °C, with a decrease of 1 °C per hour, and then cryogenically preserved for 48 h at 4 °C. This was followed by gradual cooling from 4 °C to −5 °C at a rate of 1 °C per hour, and samples were collected after 3 h at −5 °C. Then the contents of MDA, H_2_O_2_, proline and antioxidant (Peroxidase POD, Superoxide dismutase SOD, Catalase CAT) enzyme activity were determined. The transcriptional abundance of cold response genes *AtCBF* (*AtCBF1*, *AtCBF2*, *AtCBF3*) and *AtICE1* were determined. It was then cooled gradually from −5 °C to −8 °C, with a decrease of 1 °C per hour. After 12 h of treatment at −8 °C, the phenotype was observed. After cold treatment, the survival of WT and OE plants was observed at room temperature for a week.

### 4.7. Grape Calli Transformation and Treatment

Calli was induced from the grape ovary of ‘Pinot Noir’ by basic medium NN69 (2.5 μmol·L^−1^ 2,4-D + 2.5 μmol·L^−1^ NOA + 5 μmol·L^−1^ CPPU, culture in dark incubator at 25 °C). After successful induction, calli was subcultured on B_5_ medium (0.5 mg·L^−1^ NAA + 1 mg·L^−1^ KT).

The new grape calli was transferred to B_5_ solid medium and cultured for 10 days in a dark incubator at 25 °C to the calli to be infected. *Agrobacterium* solution was absorbed 200 μL and added to 50 mL LB liquid medium (containing 50 mg·L^−1^ Kanamycin and 50 mg·L^−1^ Rifampicin), cultured at 220 rpm at 28 °C to OD_600_ = 0.4–0.8. The bacterial liquid 5000 rpm was centrifuged for 10 min, then the supernatant was discarded and re-suspended in a 20 mL B_5_ liquid medium. The resuscitated bacterial liquid 5000 rpm was centrifuged for 10 min, and the supernatant was re-suspended in a B_5_ liquid medium (containing 50 mg·L^−1^ Acetosyringone). OD_600_ = 0.4 was adjusted and incubated at 28 °C for 3 h to obtain *Agrobacterium tumefaciens* suspension infection liquid.

The recombinant plasmids *VvJAZ13* were transformed into ‘Pinot Noir’ calli following the *Agrobacterium*-mediated procedure according to the method proposed by Liang et al. [61]. The grape calli was transferred to a re-suspended *Agrobacterium tumefaciens* solution to shake for 8 min, then the calli was filtered with aseptic filter paper and cultured on a solid medium of 1/2 B_5_ + 1.5% sucrose for 2 days. After culture, the calli were transferred to a B_5_ solid selective medium (containing 10 mg·L^−1^ Kanamycin, 150 mg·L^−1^ Cefamycin) and cultured in the dark environment of 28 °C for 3–4 weeks. Then the screening medium was changed until new calli were formed, and then positive calli were identified at DNA level.

WT and transgenic grape calli were treated with cold stress at 4 °C for 10 days under dark conditions and then observed for phenotypic changes. After that, we determined the content of MDA, H_2_O_2,_ and proline and the activity of antioxidant enzymes (POD, SOD, CAT). The transcriptional abundance of cold response genes *VvCBF* (*VvCBF1*, *VvCBF2*, *VvCBF3*) and *VvICE1* (*VvICE1a*, *VvICE1b*, *VvICE1c*) were determined.

### 4.8. Y2H Protein Binding Assay

The CDS sequence of *VvJAZ1*3 was cloned into pGBKT7 and fused to the DNA-binding domain to generate a bait construct. The primer sequences used are listed in supplementary Table S1. Y2H competent cells were prepared by a yeast peptone dextrose adenine (YPDA) liquid media. The recombinant plasmid was transformed into a Y2H Gold yeast strain and cultured on SD/-Trp medium at 30 °C for 3 days. The gene VvJAZ13 self-activation was detected. The potential interacting proteins were screened from the cold treatment-related AD library constructed and preserved by the Laboratory of Physiology and Biotechnology (Gansu Agricultural University of China). Screened potential protein sequences were cloned into pGADT7 and fused to activation domain sequences to generate prey construct. Yeast transformation and point-to-point verification of interaction proteins refer to the methods of Ren et al. [65].

### 4.9. Determination of Physiological Indexes of Arabidopsis and Grape Calli

The REL was measured using a model DDS307A (Leica Instruments Shanghai Co., Ltd., Shanghai, China). For subsequent data processing, refer to our previous methods [66]. H_2_O_2_, MDA, proline, and antioxidant enzyme activities (POD, SOD, CAT) were measured using a commercial ELISA kit purchased from Suzhou Comin Biotechnology Co., Ltd. (Suzhou, China) following the manufacturer’s instructions. Reaction mixtures were measured using a Multimode Reader (Spark^®^ Multifunctional micro-plate detector, TECAN, Pudong, Shanghai, China).

### 4.10. Statistical Analysis

All data were determined in three independent biological replicates for each experiment. Data analysis is based on our previous method. Data analysis was performed by one-way analysis of variance (ANOVA) using SPSS 22.0 (SPSS, Inc., Chicago, IL, USA). Duncan’s multiple range test examined whether differences were significant (*p* < 0.05). The experimental data were processed by the 2^−ΔΔCT^ method.

## 5. Conclusions

**Figure 10:**
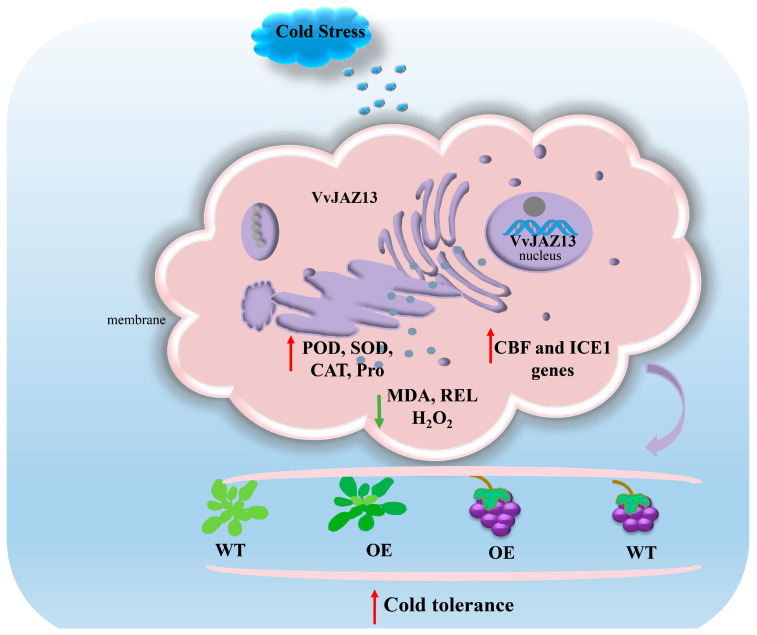
Systematic model of overexpression of *VvJAZ13* gene to improve cold tolerance of *Arabidopsis* and grape. Green and red arrows represent down and up-regulated expression levels in WT vs OE.

In conclusion, overexpression of *VvJAZ13* can increase the antioxidant enzyme activity and proline content of *Arabidopsis* and grape, decrease REL and MDA content, and increase the transcriptional abundance of cold response genes *CBF1-3* and *ICE1*, under cold stress, thereby improving the antioxidant capacity of transgenic plants and reducing membrane lipid peroxidation and accumulation of reactive oxygen species. Therefore, the overexpression of *VvJAZ13* can improve the cold resistance of *Arabidopsis* and grape (Figure 10). This study laid a theoretical foundation for further understanding the biological function and molecular mechanism of *VvJAZ13* under stress, and also provided a reference for further study of grape breeding under stress.

## Data Availability

Data is contained within the article and Supplementary Materials.

## Supplementary Materials

The following supporting information can be downloaded at https://www.mdpi.com/article/10.3390/ijms25084458/s1.
