# Supplementary material for: VvJAZ13 Positively Regulates Cold Tolerance in Arabidopsis and Grape
Source: Int J Mol Sci. 2024 Apr 18;25(8):4458. doi: 10.3390/ijms25084458 (PMC11049880; doi:10.3390/ijms25084458)
Supplement: Supplementary file 1 [file ijms-25-04458-s001.zip › ijms-2934146-supplementary.pdf]

Table S1. Primers sequence.

Primer name	primer sequence（5'→3'）	application
VvJAZ13-F	tttggagaggacacgctcgagATGACATGCAGTCTGGAAGGAAA	pART-CAM-*VvJAZ13*-
VvJAZ13-R	gcccttgctcaccatgaattcCTTTGGAGATTTGGCAGCCA	EGFP
VvJAZ13-F	aggccgaattcccggggatccttATGACATGCAGTCTGGAAGGAAA	pGBKT7-
VvJAZ13-R	ccgctgcaggtcgacggatccCTACTTTGGAGATTTGGCAGCC	*VvJAZ13*
VvJAZ13-F	CCGAATCGCTCCGCCAGAC	qRT-PCR
VvJAZ13-R	GCCGGAAACCCCAGCAGATC	qRT-PCR
VvCBF1-F	AACCCAACTGCACCATCTTC	qRT-PCR
VvCBF1-R	CATCATCCCAGCTGAATCCT	qRT-PCR
VvCBF2-F	ATGGACTTGGACCGTGAGTC	qRT-PCR
VvCBF2-R	ATCGGGAAAATTGAGGGAAG	qRT-PCR
VvCBF3-F	CCCTCATCCTCCTCTTCCTC	qRT-PCR
VvCBF3-R	TCCCCAGCTGAAGATGACTT	qRT-PCR
VvICE1a-F	GCAATGCCGGTCAGAATCAC	qRT-PCR
VvICE1a-R	AACACGAAGACGACGAGTCC	qRT-PCR
VvICE1b-F	CCAGCAAGGGTGGAGGTAAG	qRT-PCR
VvICE1b-R	AGCCTGCTGGATGTCCAATC	qRT-PCR
VvICE1c-F	TGTCTCAGAGTCAGAGCGGA	qRT-PCR
VvICE1c-R	ACCCACAAACCATCGAGGAC	qRT-PCR
VvF-box-F	tttggagaggacacgctcgagATGATAGAAACTGAAGAAGTCCAGTCTAA	pGADT7-
VvF-box-R	gcccttgctcaccatgaattcAAAAGTAGGCAAGTAGTCGTCGTCT	*VvF-box*
VvTIFY5A-F	gtgggcatcgatacgggatccttATGGAGTTTACCCCCAATCTAAGA	pGADT7-
VvTIFY5A-R	cagctcgagctcgatggatccTTAATGATTGTATGGGGACATAGCTT	*VvTIFY5A*
VvAGD13-F	gtgggcatcgatacgggatccttATGGCGACAGGAATTTCGG	pGADT7-
VvAGD13-R	cagctcgagctcgatggatccTCATGAAGAGTCAGATGATTTAATTCTTC	*VvAGD13*
VvTIFY9-F	tttggagaggacacgctcgagATGGAGAATTTGTTGGGTCTTCTC	pGADT7-
VvTIFY9-R	gcccttgctcaccatgaattcAAACAGTCCCCTGCAGCCTG	*VvTIFY9*
Vvbch1-F	gtgggcatcgatacgggatccttATGTCGAGAGCCGCCATGG	pGADT7-
Vvbch1-R	cagctcgagctcgatggatccCTAGGCCCGGAACAGCTCC	*Vvbch1*
PGADT7-F	GGAGTACCCATAGGACGTACC	pGADT7
PGADT7-R	TATCTACGATTCZTCTGCAGC	pGADT7
AtCBF1-F	GAGACGATGGTGGAAGCTATTT	qRT-PCR
AtCBF1-R	AGCATGCCTTCAGCCATATTA	qRT-PCR
AtCBF2-F	GACCTTGGTGGAGGCTATTT	qRT-PCR
AtCBF2-R	ATCCCTTCGGCCATGTTATC	qRT-PCR
AtCBF3-F	GACGTTGGTGGAGGCTATTT	qRT-PCR
AtCBF3-R	AGCATCCCTTCTGCCATATTAG	qRT-PCR
AtICE1-F	CTTCCATCCGTTGACACCTAC	qRT-PCR
AtICE1-R	CTCTAGCTTGCTGGCCTTTAG	qRT-PCR
EGFP-F	ACTATCCTTCGCAAGACC	positive identification
EGFP-R	GGTCAGCTTGCCGTAGGT	

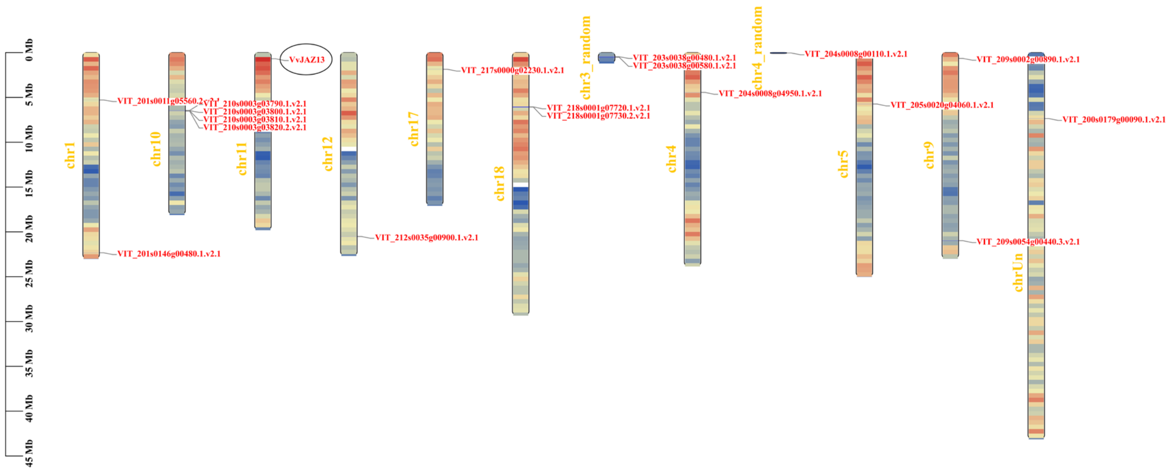

Figure S1. Chromosomal localization analysis of *VvJAZ13* gene in grape.

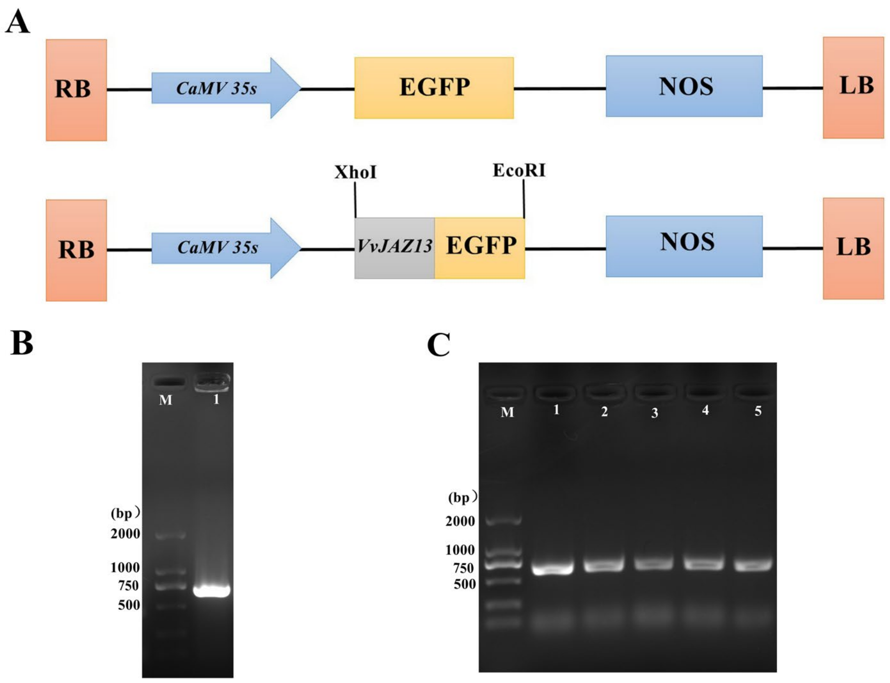

Figure S2. Cloning of *VvJAZ13* gene. (A) Empty plasmid and *VvJAZ13*-EGFP plasmid map. (B) Gene clone of *VvJAZ13*. (C) PCR detection of *Escherichia* coli. (C) PCR detection of *Agrobacterium*.

Note: M is DL 2000 Marker, 1Mel 5 is positive PCR products.

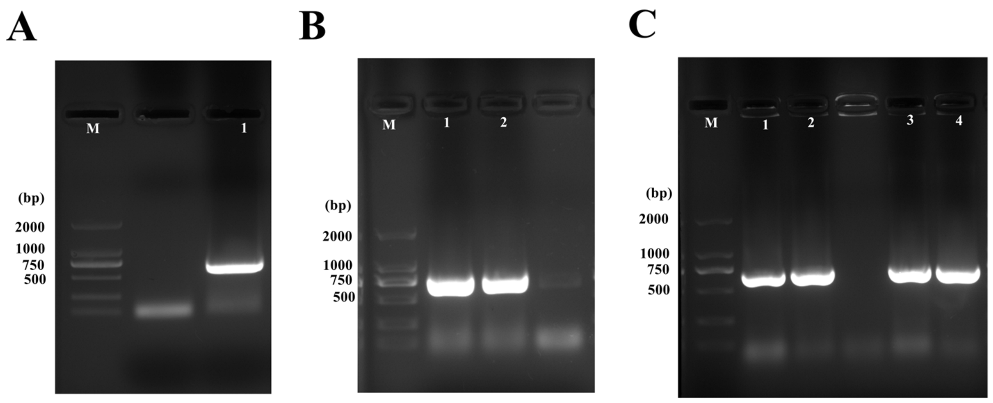

Figure S3. Cloning of VvJAZ13 gene and construction of pGBKT7-VvJAZ13 vector. (A) Gene clone of *VvJAZ13*. (B) PCR detection of *Escherichia* coli. (C) PCR detection of Y2H gold.

Note: M is DL 2000 Marker, 1Mel 5 is positive PCR products.

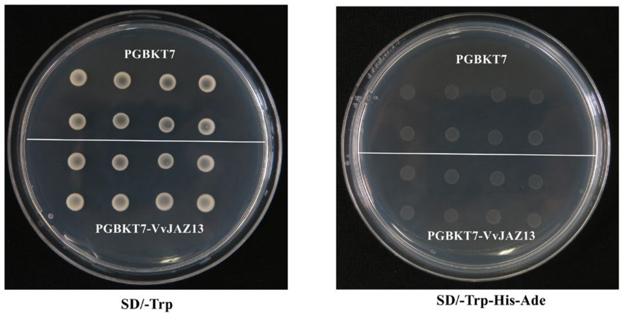

Figure S4. Transcriptional activation assays of *VvJAZ13*.

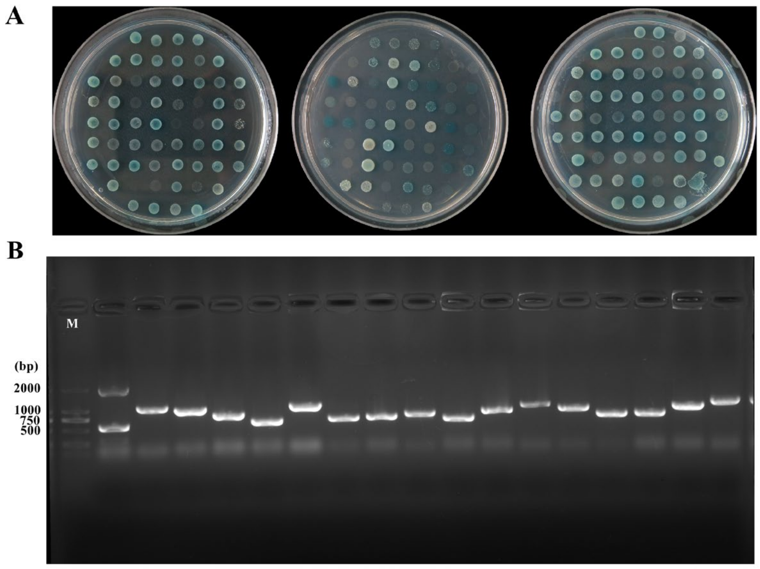

Figure S5. Screening of VvJAZ13-BD libraries. (A) VvJAZ13-BD partial library screening of cyanobacteria. (B) PCR detection plot.

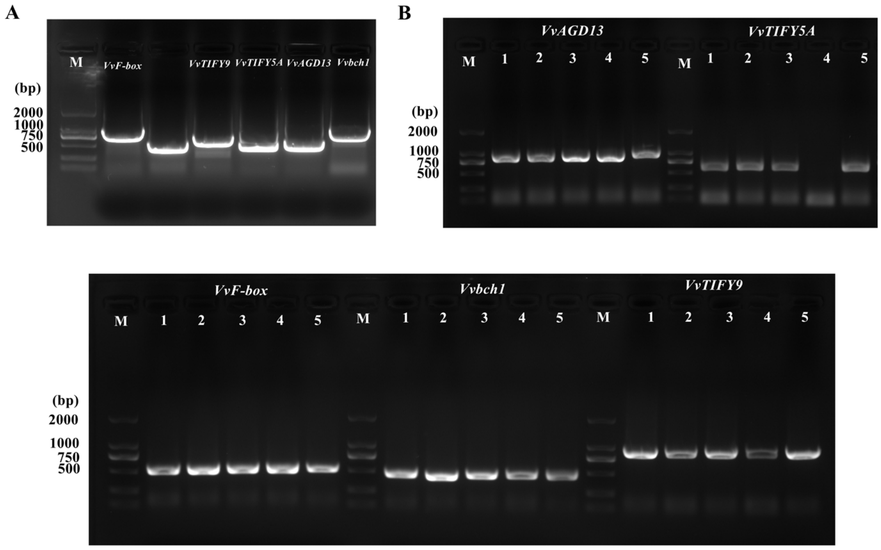

Figure S6. Cloning of *VvJAZ13* potential interacting proteins and construction of vectors.

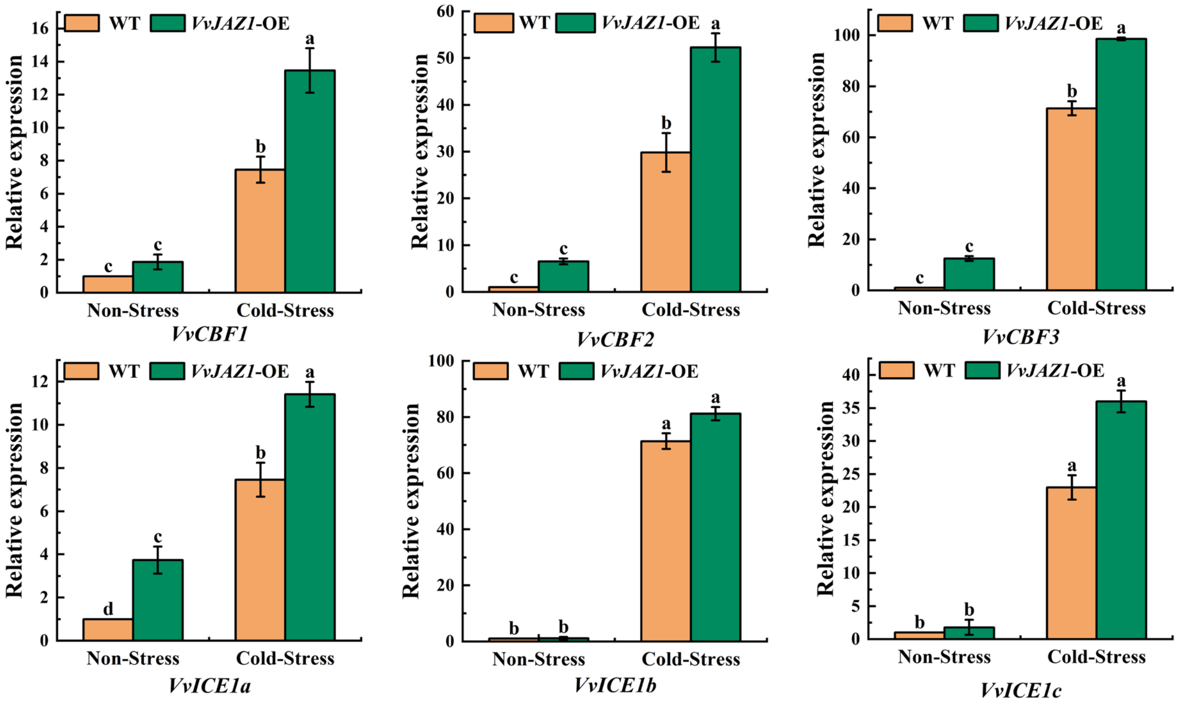

Figure S7. Expression of cold response gene *VvCBF1*, *VvCBF2*, *VvCBF3*, *VvICE1a*, *VvICE1b* and *VvICE1c* in transient transformation of *VvJAZ13* grape leaves.

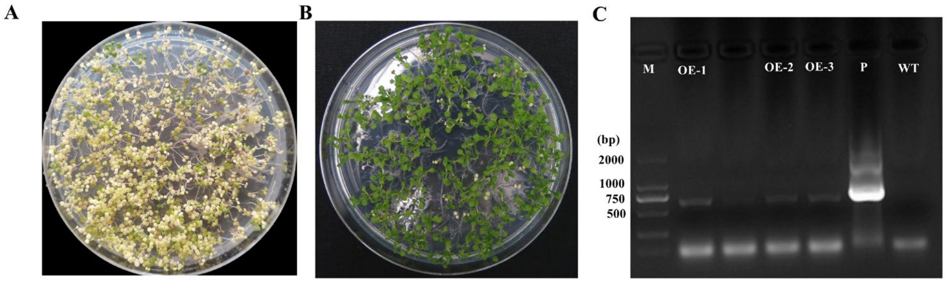

Figure S8. Identification of *VvJAZ13* transgenic *Arabidopsis*. (A) Screening of T_0 *Arabidopsis* seeds in MS medium containing Kana resistance. (B) Screening of T_2 homozygous *Arabidopsis* seeds in in MS medium containing Kana resistance. (C) The positive detection of *Arabidopsis*.
